# Phosphorylation and regulation of group II metabotropic glutamate receptors (mGlu2/3) in neurons

**DOI:** 10.3389/fcell.2022.1022544

**Published:** 2022-11-03

**Authors:** Li-Min Mao, Nirav Mathur, Tayyibah Mahmood, Sri Rajan, Xiang-Ping Chu, John Q. Wang

**Affiliations:** ^1^ Department of Biomedical Sciences, School of Medicine, University of Missouri-Kansas City, Kansas City, MO, United States; ^2^ Department of Anesthesiology, School of Medicine, University of Missouri-Kansas City, Kansas City, MO, United States

**Keywords:** mGlu, glutamate, phosphorylation, PKA, PKC, casein kinase, phosphatase

## Abstract

Group II metabotropic glutamate (mGlu) receptors (mGlu2/3) are Gαi/o-coupled receptors and are primarily located on presynaptic axonal terminals in the central nervous system. Like ionotropic glutamate receptors, group II mGlu receptors are subject to regulation by posttranslational phosphorylation. Pharmacological evidence suggests that several serine/threonine protein kinases possess the ability to regulate mGlu2/3 receptors. Detailed mapping of phosphorylation residues has revealed that protein kinase A (PKA) phosphorylates mGlu2/3 receptors at a specific serine site on their intracellular C-terminal tails in heterologous cells or neurons, which underlies physiological modulation of mGlu2/3 signaling. Casein kinases promote mGlu2 phosphorylation at a specific site. Tyrosine protein kinases also target group II receptors to induce robust phosphorylation. A protein phosphatase was found to specifically bind to mGlu3 receptors and dephosphorylate the receptor at a PKA-sensitive site. This review summarizes recent progress in research on group II receptor phosphorylation and the phosphorylation-dependent regulation of group II receptor functions. We further explore the potential linkage of mGlu2/3 phosphorylation to various neurological and neuropsychiatric disorders, and discuss future research aimed at analyzing novel biochemical and physiological properties of mGlu2/3 phosphorylation.

## Introduction

Two classes of cell-surface and membrane-bound glutamate receptors are expressed in the mammalian brain. Ionotropic glutamate (iGlu) receptors are a family of ligand-gated ion channels that mediate fast excitatory synaptic transmission (Traynelis et al., 2010). Another class of glutamate receptors are G protein-coupled receptors (GPCR), i.e., metabotropic glutamate (mGlu) receptors which include a total of eight mGlu subtypes (mGlu1-8). Three functional mGlu groups (I-III) are subdivided from eight subtypes based on sequence homology, signaling transduction, and pharmacological properties (Niswender and Conn, 2010). Group II mGlu receptors are comprised of mGlu2 and mGlu3 subtypes and constitute a subgroup of mGlu receptors that are canonically linked to the Gαi/o heterotrimeric G proteins. Similar to other mGlu receptors, mGlu2/3 function primarily as dimers and are enriched at synaptic sites. As opposed to group I receptors (mGlu1/5) that are mostly postsynaptic, group II receptors are abundantly distributed on presynaptic axonal terminals (Niswender and Conn, 2010; Nicoletti et al., 2011). By responding to excessive synaptic glutamate or glutamate released from astrocytes, mGlu2/3 act as autoreceptors on glutamatergic terminals or heteroreceptors on other phenotypic terminals to inhibit presynaptic release of glutamate or other transmitters, respectively. As such, mGlu2/3 receptors have drawn increasing attention as robust regulators of synaptic transmission and plasticity and potential targets for developing novel pharmacotherapies for various brain disorders (reviewed in Muguruza et al., 2016; Chaki, 2017; Maksymetz et al., 2017; Mazzitelli et al., 2018; Sebastianutto and Cenci, 2018).

Protein phosphorylation is one of important posttranslational modifications that actively regulate expression, distribution, and function of modified proteins under normal conditions or in response to changing synaptic input. Glutamate receptors are among a pool of synaptic proteins that are subject to vigorous regulation by a phosphorylation-dependent mechanism (Mao et al., 2011; Diering and Huganir, 2018; Purkey and Dell’Acqua, 2020). Early extensive studies have well documented the phosphorylation of iGlu receptors (Wang et al., 2014; Lussier et al., 2015; Diering and Huganir, 2018). Phosphorylation of group I mGlu receptors has also been characterized in a large number of studies *in vitro* and in neurons (Kim et al., 2008; Mao et al., 2008; Suh et al., 2018). In addition to iGlu and group I receptors, available evidence supports group II receptors as efficient substrates of protein kinases. Specific amino acid residues undergoing phosphorylation have been identified in intracellular domains of recombinant group II receptors transfected in heterologous expression cells. Such site-specific phosphorylation was also confirmed in native group II receptors in the adult animal brain *in vivo*. Several synapse-enriched protein kinases are involved in mGlu2/3 phosphorylation and/or regulation, including protein kinase A (PKA), protein kinase C (PKC), and G protein-coupled receptor kinases (GRK). Noticeably, mGlu2/3 phosphorylation is a regulated event and is sensitive to changing cellular and synaptic signals. As such, mGlu2/3 phosphorylation levels are altered in an animal model of a neuropsychiatric disease, reflecting a significant adaptive event in the remodeling of excitatory synaptic transmission and plasticity critical for the pathogenesis and symptomatology of the disease.

## Group II metabotropic glutamate receptors

The GRM2 and GRM3 genes encode human mGlu2 and mGlu3 receptor proteins, respectively [mGlu2 receptor accession numbers: NP_000830 (human), NP_001099181 (rat), and NP_001153825 (mouse); mGlu3 receptor accession numbers: NP_000831 (human), NP_001099182 (rat), and NP_862898 (mouse)] (Tanabe et al., 1992; Flor et al., 1995; Nicoletti et al., 2011). The amino acid sequence of the mGlu2 receptor shares approximately 70% homology with the mGlu3 receptor (Pin and Duvoisin, 1995). While no evidence for alternative splicing of GRM2 was observed, alternative splicing of GRM3 resulted in three transcript (mRNA) splice variants different from the canonical full-length GRM3 transcript: GRM3Δ2 (lacking exon 2), GRM3Δ4 (lacking exon 4), and GRM3Δ2Δ3 (lacking exons 2 and 3) (Sartorius et al., 2006). The GRM3Δ4 transcript showed translatability and was translated to a protein splice variant. i.e., a truncated mGlu3 receptor (GRM3Δ4), in the human brain.

**FIGURE 1:**
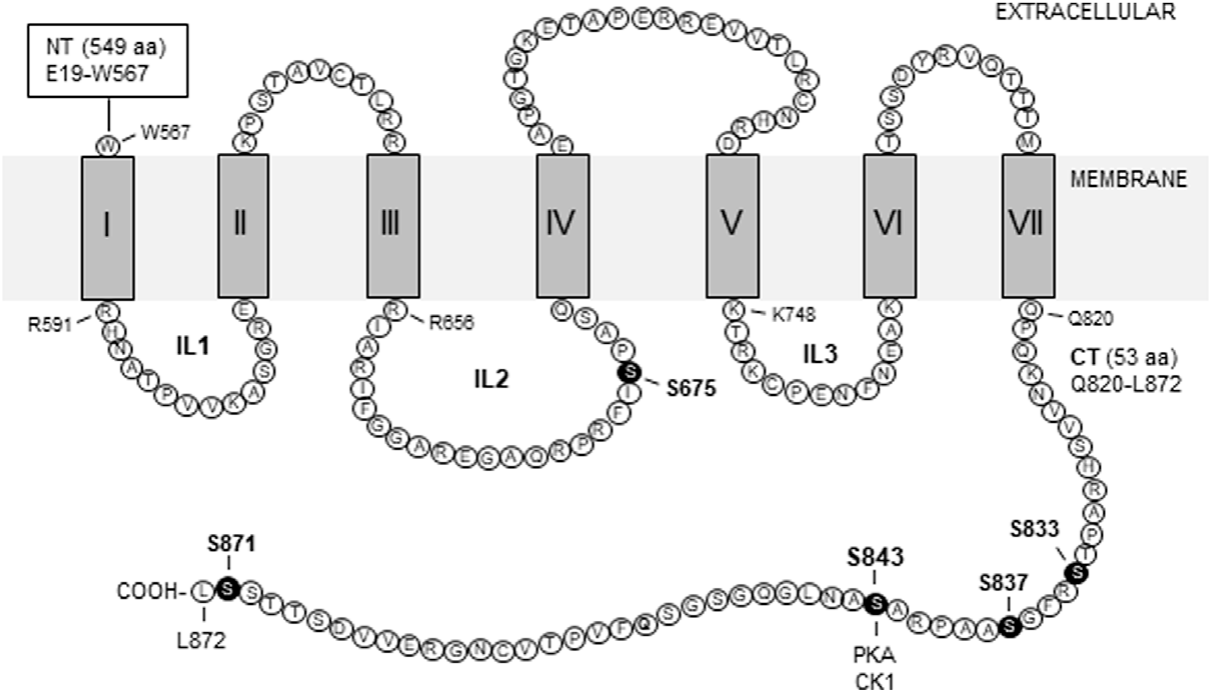
Schematic illustration of transmembrane topology of rat mGlu2 receptors. The illustrated regions include seven transmembrane domains (I-VII) and four intracellular domains, i.e., the intracellular loop 1 (IL1), IL2, IL3, and C-terminus (CT). Amino acid sequences of intracellular domains are labeled by the single letter code (accession #: NP_001099181/NCBI; P31421/UniProtKB). Note that all intracellular domains contain serine and/or threonine but not tyrosine residues. Solid circles indicate the serine sites where variable levels of phosphorylation have been detected. Other abbreviations: CK1, casein kinase 1; NT, N-terminus; PKA, protein kinase A.

Group II receptors have a traditional membrane topology for a class C GPCR: A large extracellular N-terminus, an intracellular C-terminus (CT), and seven transmembrane domains which give rise to three intracellular loops. A schematic topology of the rat mGlu2 receptor is illustrated in Figure 1 with labeled amino acid sequences for intracellular domains (Tanabe et al., 1992). The CT domain is a key region where the receptor forms dynamic signaling complexes with a number of synaptic and cytoplasmic proteins and harbors site-specific phosphorylation balanced by a fine kinase-phosphatase cooperation.

mGlu2/3 receptors are diffusely expressed in broad regions of the central nervous system (Ohishi et al., 1993, 1994; Neki et al., 1996; Petralia et al., 1996; Luján et al., 1997; Tamaru et al., 2001; Wright et al., 2013), with mGlu3 being also seen in B lymphoblasts (Sartorius et al., 2006). Studies have shown that mGlu2 expression seems to be restricted to neurons, whereas mGlu3 was found in neurons as well as glial cells (Tanabe et al., 1993; Ohishi et al., 1998). A moderate-to-high level of mGlu2/3 expression in the limbic system, including the prefrontal cortex, amygdala, hippocampus, thalamus, and striatum, is noteworthy as these regions support emotion, learning and memory, motivation, reward, and behavior. At the subsynaptic level, mGlu2/3 receptors are mostly presynaptic and reside in an area outside of the active zone of axonal terminals, while both subtypes may also be postsynaptic (Neki et al., 1996; Lujan et al., 1997; Tamaru et al., 2001). Of note, mGlu2-like immunoreactive neurons in the striatum are large aspiny neurons which may correspond to cholinergic interneurons (Ohishi et al., 1998).

As Gαi/o-coupled receptors, mGlu2/3 receptors upon activation inhibit adenylyl cyclase, leading to a decrease in downstream cAMP production and PKA activity (Niswender and Conn, 2010). In addition to this canonical signaling pathway, mGlu2/3 receptors activate the mitogen-activated protein kinase (MAPK)/extracellular signal-regulated kinase pathway, probably *via* Gβγ release (Phillips et al., 1998; Ferraguti et al., 1999; Wang et al., 2006). mGlu2/3 also inhibit voltage-sensitive Ca^2+^ channels and activate K^+^ channels (Niswender and Conn, 2010; Nicoletti et al., 2011). As presynaptic autoreceptors, mGlu2/3 dynamically inhibit glutamate release to maintain the homeostasis of excitatory synaptic transmission. Besides, mGlu2/3 function as heteroreceptors to negatively modulate nonglutamatergic transmitter release, such as inhibitory amino acids, monoamines, and neuropeptides (Cartmell and Schoepp, 2000). Both receptors play a role in the modulation of synaptic plasticity, particularly in the induction of long-term depression, a common form of synaptic plasticity (Bellone et al., 2008; Nicoletti et al., 2011). Although comprehensive understanding of physiology and pharmacology remains elusive, emerging evidence suggests that different mGlu subtypes could naturally form heterodimers. mGlu2/4 heterodimers are the most studied pair among heterodimeric mGlu receptors to date. Both subtypes could form mGlu2/4 heterodimers in brain cells *in vivo*, in addition to their prime homodimers (Doumazane et al., 2011; Yin et al., 2014; Moreno-Delgado et al., 2017; Pin JP et al., 2019; Meng et al., 2022).

## Phosphorylation of group II metabotropic glutamate receptors by protein kinase A

PKA has been most thoroughly investigated for its role in direct phosphorylation of group II mGlu receptors. Early pharmacological studies showed that the adenylyl cyclase activator, forskolin, suppressed group II receptors in their ability to presynaptically inhibit glutamatergic synaptic transmission in the hippocampus (Kamiya and Yamamoto 1997; Maccaferri et al., 1998), although not in the subthalamus (Shen and Johnson, 2003). Forskolin also inhibited the mGlu2/3 function at the medial perforant path-dentate granule cell synapse, a synapse where synaptic inhibition is likely mediated by the mGlu2 subtype (Neki et al., 1996; Shigemoto et al., 1997). Thus, activation of adenylyl cyclase and the downstream cAMP pathway is considered to exert a negative regulation of mGlu2/3 activity. Since the forskolin-cAMP pathway is less likely to inhibit mGlu2/3 by simply overcoming the reduction of intracellular cAMP levels induced by the mGlu2/3 agonist (Schaffhauser et al., 2000), forskolin was thought to trigger a molecular event that has a direct impact on mGlu2/3 receptors.

**FIGURE 2:**
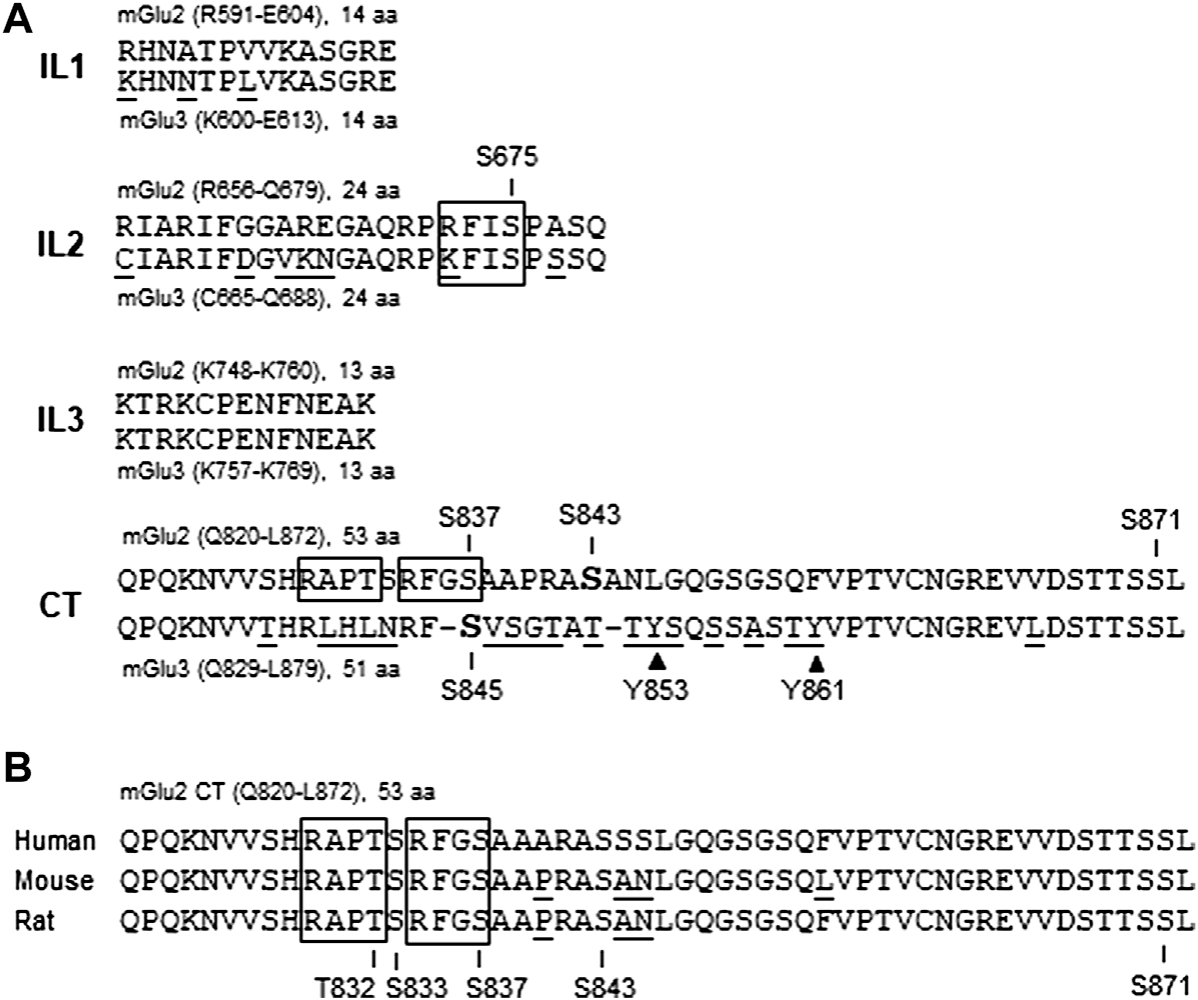
Alignment of amino acid sequences of intracellular domains between mGlu2 and mGlu3 subtypes and among three species. **(A)** Sequence alignment of intracellular domains between mGlu2 *versus* mGlu3 receptors. Intracellular domains include the intracellular loop 1 (IL1), IL2, IL3, and C-terminus (CT). Gaps (–) are inserted in the mGlu3 CT region to achieve maximum homolog. Note that the mGlu3 CT region contains two tyrosine residues (Y853 and Y861) indicated by solid triangles. **(B)** Sequence alignment of the CT regions among human, mouse, and rat mGlu2 orthologs. Note that the S843 residue is conserved in the three species. Underlined amino acids denote differences in sequence alignment. Open boxes indicate CaMKII consensus phosphorylation sequences.

To elucidate the precise molecular mechanism underlying the forskolin action in inhibiting mGlu2 receptors, Schaffhauser et al. (2000) explored possible phosphorylation of mGlu2 receptors in response to forskolin stimulation. They found that mGlu2 was a sufficient substrate of a cAMP-dependent kinase, i.e., PKA. In detail, the cAMP analog (8-bromo-cAMP) increased phosphorylation of mGlu2 receptors in cerebellar granule cells. Applying PKA also elevated the amount of phospho-mGlu2/3 proteins in hippocampal neurons. Further *in vitro* experiments with purified recombinant proteins containing individual intracellular domains revealed that PKA strongly phosphorylated the mGlu2 CT tail (residues Q820-L872) but not the first intracellular loop. Between two sites (S837 and S843) similar to the PKA consensus phosphorylation motif R/K-R/K-X-S/T (where X represents any amino acid) in mGlu2 CT (Figure 2), S843 was the primary residue phosphorylated by PKA. The fact that S843 is conserved among humans, rats, and mice is noteworthy (Figure 2B). In addition to the CT tail, a serine site (S675) on the second intracellular loop of mGlu2 receptors (Figures 1, 2A) was demonstrated to be a minor site slightly phosphorylated by PKA (Schaffhauser et al., 2000).

It is important to point out that the PKA-mediated phosphorylation is not restricted to mGlu2 receptors. A serine site (S845) in the mGlu3 CT tail (Figure 2) was also phosphorylated by PKA (Cai et al., 2001; Flajolet et al., 2003). In adult rat brains *in vivo*, serine phosphorylation was seen on endogenous mGlu2/3 receptors immunoprecipitated from the striatum and prefrontal cortex (Xi et al., 2002). Using a phospho- and site-specific antibody against the S843-phosphorylated form of mGlu2 receptors, phosphorylation of native mGlu2 receptors was detected in the mouse prefrontal cortex (Murat et al., 2019). Of note, during searching for a source receptor from which the cAMP-PKA pathway is activated to downregulate mGlu2/3, Gαs-coupled β-adrenergic receptors were found to activate PKA and thus inhibit mGlu2/3 receptors at hippocampal synapses (Cai et al., 2001).

Phosphorylation of mGlu2/3 by PKA has significant physiological consequences. Mutation of mGlu2 S843 to alanine (S843A) prevented phosphorylation at this PKA site (Schaffhauser et al., 2000). This mutation also abolished the ability of the cAMP analog to inhibit the mGlu2-mediated response, while the same mutation at S675 on the second intracellular loop that was slightly phosphorylated by PKA had no effect. These results indicate that S843 phosphorylation is essential for PKA to modulate mGlu2 function. Further studies showed that phosphorylation of S843 inhibited G protein coupling to mGlu2 receptors, which may serve as a mechanism to underlie PKA’s action in regulating the receptor in a negative fashion (Schaffhauser et al., 2000).

## Regulation of group II metabotropic glutamate receptors by G protein-coupled receptor kinases

In addition to second messenger-dependent protein kinases (PKA, PKC, etc.), GRKs phosphorylate and thus regulate GPCRs. GRKs phosphorylate the ligand-activated GPCRs at serine and threonine sites, which facilitates the binding of an arrestin protein to the active receptor. Arrestin binding then interdicts the receptor-G protein coupling, resulting in the agonist-promoted homologous desensitization, one of the most noticeable roles of GRKs. Group I mGlu receptors are among GPCRs that are readily phosphorylated and regulated by several members of the GRK family (Mao et al., 2008). mGlu3 receptors are also a target of GRKs. Iacovelli et al. (2009) found that the mGlu3 agonist inhibited cAMP formation and activated MAPKs, which were fully desensitized by GRK2, GRK3, or overexpression of β-arrestin1 in transfected HEK293 cells. The agonist-induced desensitization of mGlu3 also existed in mouse cortical slices. Thus, GRKs possess the ability to modulate mGlu3 *via* a homologous desensitization process.

How GRKs modulate mGlu3 receptors is unclear at present. Traditionally, GRKs could phosphorylate a receptor to initiate a GRK-arrestin process to desensitize the receptor. Alternatively, GRKs could achieve their roles by regulating receptor/G protein interactions without requiring GRK-mediated phosphorylation as has been seen in mGlu1a receptors (Dhami et al., 2002, 2004; Dhami and Ferguson, 2006) or by phosphorylating non-receptor substrates. Future experiments will need to clarify whether the mGlu3 receptor is a direct substrate of GRKs and if so which amino acid(s) are actually phosphorylated by GRKs. Of note, a rigidly defined phosphorylation consensus motif has not been established for GRKs as opposed to many second messenger-dependent protein kinases.

The regulation of mGlu2 receptors by GRKs is signal dependent. Like mGlu3, the mGlu2-triggered MAPK signaling pathway was desensitized by GRK2 (Iacovelli et al., 2009). However, unlike mGlu3, the cAMP signaling induced by mGlu2 receptors was not desensitized by the GRK/β-arrestin1 apparatus in transfected HEK293 cells. The resistance of the mGlu2-cAMP signaling to GRK-dependent homologous desensitization was confirmed in native mGlu2 receptors *in vivo* as the mGlu2/3 agonist-induced inhibition of cAMP responses was desensitized in wild-type and mGlu2−/− mice but not in mGlu3−/− mice. Moreover, such resistance was consistently seen in CHO and C6 glioma expression cells and in neurons (Lennon et al., 2010). Regarding the inhibition of field excitatory postsynaptic potentials, the agonist-induced mGlu2 activity exhibited no rapid agonist-dependent homologous desensitization at medial perforant path synapses in the rat hippocampus (Schaffhauser et al., 2000). Altogether, the mGlu2 function (mainly cAMP signaling) is resistant to the homologous regulation involving GRKs and is therefore suggested to be regulated by a heterologous mechanism involving second messenger-dependent protein kinases.

## Phosphorylation of group II metabotropic glutamate receptors by other protein kinases

iGlu receptors (Wang et al., 2014; Lussier et al., 2015; Diering and Huganir, 2018), group I mGlu receptors (Kim et al., 2008; Mao et al., 2008; Suh et al., 2018), and group III mGlu receptors (Airas et al., 2001) are all regulated by PKC. Group II mGlu receptors are no exception based on pharmacological evidence. For example, a PKC activator inhibited the mGlu2/3-mediated responses in corticostriatal co-cultures (Tyler and Lovinger, 1995) and the presynaptic mGlu receptor-mediated inhibition of corticostriatal synapses in striatal slices (Swartz et al., 1993). Similar results were found at excitatory synapses in the hippocampus (Kamiya and Yamamoto, 1997; Macek et al., 1998; Lennon et al., 2010) and other brain regions (Shen and Johnson, 2003; Huang et al., 2007). These results indicate that the major presynaptic inhibitory function of mGlu2/3 receptors in multiple brain regions is subject to the negative regulation by PKC. Considering that mGlu2 receptors are resistant to the agonist-induced homologous desensitization, PKC may be an alternative second messenger-dependent kinase responsible for the agonist-independent heterologous desensitization of mGlu2 receptors (Lennon et al., 2010). The finding that PKC activation induced mGlu2 internalization supports this notion. Mechanistically, PKC could inhibit mGlu2/3 function directly by phosphorylating mGlu2/3 receptors or indirectly by phosphorylating any other signaling and effector proteins that are involved in modulating mGlu2/3 receptors. It is possible that mGlu2/3 receptors contain PKC-sensitive phosphorylation site(s), although the direct PKC-phosphorylated site(s) on mGlu2/3 receptors have not been reported to be mapped to our knowledge.

Similar to PKA that phosphorylates S843 on mGlu2 receptors (see above), casein kinase 1 (CK1) also promotes S843 phosphorylation. Murat et al. (2019) recently found that active CK1 was required for the S843 phosphorylation induced by a unique positive crosstalk between mGlu2 and 5-HT2A receptors at the receptor level. In HEK293 cells and mouse prefrontal cortical neurons expressing both mGlu2 and 5-HT2A receptors, the mGlu2 agonist-induced S843 phosphorylation as detected by using a newly generated phospho- and site-specific antibody was prevented by antagonism of 5-HT2A receptors, whereas the 5-HT2A agonist-stimulated S843 phosphorylation was abolished by mGlu2 antagonism. CK1 (likely the CK1ε isoform) appears to be responsible for promoting S843 phosphorylation in response to mGlu2 activation since the CK1 inhibitor D4476 and silencing CK1ε expression with short interfering RNAs abolished the mGlu2 agonist-induced S843 phosphorylation, while the PKA inhibitor KT5720 did not. Of note, D4476 did not alter the low level of S843 phosphorylation seen in HEK293 cells expressing mGlu2 receptors alone.

Besides serine and threonine, tyrosine represents a site accepting phosphorylation in group II receptors. Orlando et al. (2002) utilized an anti-phosphotyrosine antibody to immunoprecipitate a pool of tyrosine-phosphorylated proteins from adult rat striatal tissue. Using an anti-mGlu2/3 antibody, they then visualized the existence of an mGlu2/3 immunoreactive band in the tyrosine-phosphorylated protein pool, indicating that striatal group II receptors are tyrosine-phosphorylated in the basal state. As to accurate tyrosine site(s) phosphorylated by a tyrosine kinase, two tyrosine residues are notably situated on mGlu3 CT (Y853 and Y861) (Figure 2). Meanwhile, there is no tyrosine on intracellular loops (IL1-3) of mGlu3 receptors and on all intracellular domains of mGlu2 receptors. Thus, tyrosine phosphorylation likely occurs in the mGlu3 CT region. It will be intriguing to carry out future studies to identify the precise tyrosine site(s) and the responsible tyrosine kinase(s) in relation to potential physiological implications of tyrosine phosphorylation in regulating expression and function of mGlu3 receptors.

## Dephosphorylation of metabotropic glutamate receptors by protein phosphatase

The dynamic regulation of phosphorylation status of a given protein requires subtle interactions between protein kinases and PPs. Thus, mGlu2/3 phosphorylation is reasoned to be tightly modulated by a dephosphorylation process involving PPs. In support of this assumption, Flajolet et al. (2003) found that recombinant PP2C bound to the mGlu3 CT fragment (P830-L879) *in vitro*. Native PP2C also bound to mGlu3 CT in rat brain cells. Among four brain PP2C isoforms (α, β, γ, and δ) surveyed, all of them bound to mGlu3 CT, with PP2Cβ showing the highest affinity. Among the CT regions of all mGlu subtypes (mGlu1-8), positive binding of PP2Cα was detected only with mGlu3, indicating that the highly specific nature of the PP2C-mGlu3 interaction. A 20-amino acid region (836–855) in the middle of mGlu3 CT constitutes a minimal domain for the sufficient binding to PP2Cα. This region is noticeably not conserved in mGlu2 CT, even though the amino acid sequences of the group II receptors (i.e., mGlu2 *versus* mGlu3) are highly homologous in the proximal and distal regions of CT tails (Figure 2A).

**FIGURE 3:**
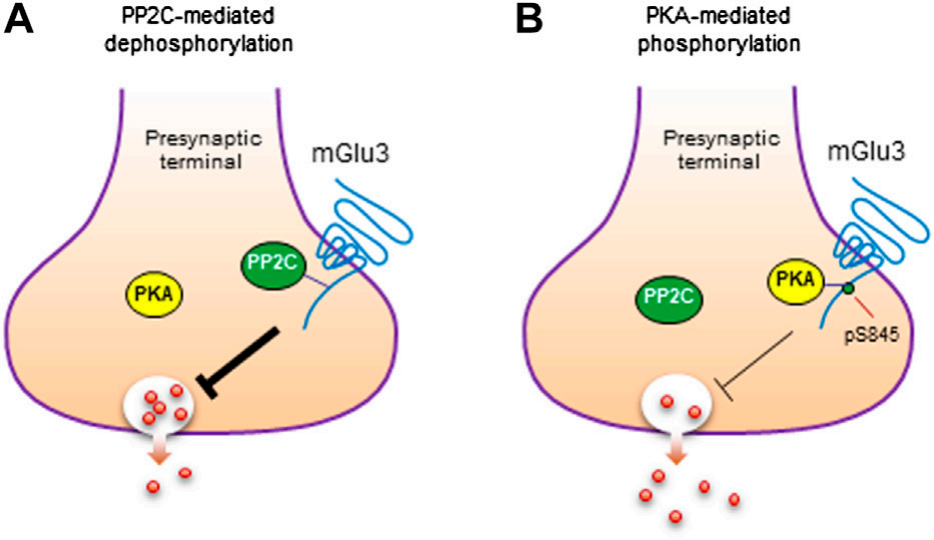
Phosphorylation and dephosphorylation of mGlu3 receptors. **(A)** Dephosphorylation of mGlu3 receptors by PP2C. PP2C dephosphorylates mGlu3 receptors at a specific serine residue (S845) in the C-terminus (CT) region of the receptor. **(B)** Phosphorylation of mGlu3 receptors by PKA. PKA phosphorylates mGlu3 receptors at S845 and increases the level of S845-phosphorylated (pS845) mGlu3 receptors. Such phosphorylation inhibits the PP2C association with mGlu3 CT and may exert an inhibitory control over the presynaptic function of mGlu3 receptors.

Given the binding of PP2C to mGlu3, PP2C may carry out a function to dephosphorylate the receptor. In fact, PP2C dephosphorylated mGlu3 receptors at a PKA site (S845), while other serine/threonine phosphatases (PP1, PP2A, and PP2B) did not (Flajolet et al., 2003). Thus, the mGlu3 receptor serves as a direct and specific substrate of PP2C. It was further observed that phosphorylation of S845 by PKA inhibited the binding of PP2C to mGlu3 CT. Moreover, the interaction of mGlu3 with PP2C inhibited PP2C phosphatase activity. These findings collectively imply an attractive model in which PKA and PP2C interplay to kinetically control the phosphorylation level of mGlu3 at S845 (Figure 3; Flajolet et al., 2003). In detail, under basal conditions, PP2C preferentially binds to a subset of mGlu3 receptors that are unphosphorylated at S845. This binding inhibits enzymatic activity of PP2C and stores PP2C within a subcellular microdomain. In response to activation of adenylyl cyclase and downstream PKA by a variety of transmitters or modulators, activated PKA enhances the steady-state phosphorylation level of mGlu3 at S845. The enhanced S845 phosphorylation could thereby dissociate PP2C from mGlu3 CT and remove the mGlu3-PP2C interaction-induced inhibition of PP2C activity. The PP2C with recovered phosphatase activity could then dephosphorylate the phospho-S845 site, resulting in a transient and reversible phosphorylation event at mGlu3 S845, although released PP2C may also act on other local substrates. After the dephosphorylation of S845, a reassociation of PP2C with mGlu3 CT is promoted to fulfill a dynamic phosphorylation-dephosphorylation cycle.

## Conclusion

Significant progress has been made in both pharmacological and biochemical studies aimed to explore and characterize the phosphorylation and regulation of presynaptic group II mGlu receptors by various protein kinases. Consistent pharmacological evidence supports a pivotal role of PKA in inhibiting mGlu2/3-mediated responses in a variety of functional assays both *in vitro* and *in vivo*. GRKs and PKC are also among presynaptic kinases that vigorously regulate mGlu2 and/or mGlu3 receptors. The pharmacologically-defined roles of kinases have been well validated by the ability of kinases to directly phosphorylate mGlu2/3 receptors. In recombinant and native mGlu2/3 proteins respectively expressed in heterologous cells and animal brains, PKA phosphorylates a conserved serine site on mGlu2/3 CT tails. Like serine and threonine, tyrosine-phosphorylated group II proteins (likely mGlu3) were found in the striatum. Phosphorylation of mGlu2/3 receptors is functionally relevant. For instance, the PKA-mediated phosphorylation of mGlu2/3 receptors negatively modulates the presynaptic inhibitory function of mGlu2/3 by reducing the coupling efficiency of G proteins to the receptors. In addition to protein kinases, PP2C specifically binds to mGlu3 but not mGlu2 receptors. This supports existence of the functional PP2C-PKA interaction controlling phosphorylation of mGlu3 receptors. Overall, mGlu2/3 phosphorylation represents an important posttranslational modification that regulates responses of mGlu2/3 to changing synaptic input and presynaptically maintains homeostasis of excitatory synaptic transmission.

It is apparent that preclinical research on phosphorylation biology of group II receptors is at its early stage. So far, many studies have focused on a pharmacological aspect. Knowledge of detailed biochemical properties and physiology of group II receptor phosphorylation is largely lacking at present. Thus, a comprehensive and in-depth understanding of group II receptor phosphorylation requires a series of new endeavors in advanced future studies. First, several potential phosphorylation sites (serine, threonine, and tyrosine) in mGlu2/3 intracellular domains (Figure 2) have yet to be charted to any kinases or phosphatases, even though not all of them are expected to be phosphorylation sites. In fact, analysis with nano-flow liquid chromatography coupled with high resolution tandem mass spectrometry has identified four phosphorylated residues (S833, S837, S843, and S871) on the CT domain of transfected mGlu2 receptors in HEK293 cells (Murat et al., 2019). S837 is particularly intriguing as it falls within an R/K-X-X-S/T region, a consensus sequence for phosphorylation by Ca2+/calmodulin-dependent protein kinase II (CaMKII) (Figure 2). On the other hand, additional kinases and phosphatases may join those discussed in this review to regulate the receptors *via* a phosphorylation-dependent mechanism. Second, given that different kinases may act on the same phosphorylation site, robust crosstalk may occur among kinases to determine precise signaling and physiological outcomes. Similarly, crosstalk could occur at additional levels, such as between kinases and phosphatases and between phosphorylation and other types of posttranslational modifications. Third, two group II subtypes may have distinguishable properties in their phosphorylation reactions. Indeed, PP2C bound to and dephosphorylated only mGlu3 receptors (Flajolet et al., 2003). The mGlu3 CT contains tyrosine residues, while the mGlu2 CT does not. Additionally, the mGlu2 CT contains CaMKII consensus motifs, which are conserved in humans, mice, and rats, while the mGlu3 CT does not (Figure 2). Fourth, astrocytes represent a major cell population in the central nervous system. The understanding of astrocytes has recently changed from a neuron-supporting cell type to an active player in multifaceted glia-neuronal and glia-synaptic interactions (Dallerac et al., 2018). mGlu3 receptors in addition to mGlu5 receptors are noticeably expressed in astrocytes and act to increase glutamate uptake by regulating excitatory amino acid transporters (Tanabe et al., 1992; Schools and Kimelberg, 1999; Aronica et al., 2003). It is likely that the glial mGlu3 receptor is subject to phosphorylation and its phosphorylation status is linked to the regulation of receptor function and excitatory synapses. Future studies can aim to investigate these topics. Finally and more importantly, protein phosphorylation plays broad roles in regulating clustering, dimerization, trafficking, anchoring, turnover, signaling, and functions of modified receptors. As a result, abnormal activity of mGlu2/3 phosphorylation is of significant clinical implications. The phosphorylation status of presynaptic mGlu2/3 receptors in relevant brain regions may undergo adaptive changes during the development of various neurological and neuropsychiatric disorders. These long-term changes may contribute to the remodeling of excitatory synaptic transmission and plasticity critical for the pathogenesis and symptomatology of chronic brain disorders. In fact, mGlu2/3 receptors have been linked to anxiety, schizophrenia, depression, chronic pain, drug addiction, and neurodegenerative disorders (Nicoletti et al., 2011; Planas-Fontanez et al., 2020; Abd-Elrahman et al., 2021; Li et al., 2022; Mazzo et al., 2022; Turati et al., 2022). Emerging evidence shows that the serine phosphorylation state of mGlu2/3 monomers was elevated in the rat nucleus accumbens and prefrontal cortex following repeated administration of cocaine (Xi et al., 2002). Together, mGlu2/3 receptors may serve as a biomarker of brain disorders and a promising target for developing a new generation of therapeutic drugs.

Methodologically, the above mentioned future studies will be facilitated by recently established tools and approaches, including 1) orthosteric or allosteric agents with high selectivity for the individual mGlu2 or mGlu3 subtype, 2) mGlu2 or mGlu3 knockout mice or mGlu2/3 double knockout mice (Wright et al., 2013), 3) mGlu2 or mGlu3 subtype-specific antibodies validated in knockout mice, 4) mGlu2 nanobodies (Scholler et al., 2017) and nanobody-based biosensors (Meng et al., 2022), and 5) phospho- and site-specific antibodies.
